# Mechanism of Action of Bu-Fei-Yi-Shen Formula in Treating Chronic Obstructive Pulmonary Disease Based on Network Pharmacology Analysis and Molecular Docking Validation

**DOI:** 10.1155/2020/9105972

**Published:** 2020-11-26

**Authors:** Longchuan Wu, Yu Chen, Jiao Yi, Yi Zhuang, Lei Cui, Chunhui Ye

**Affiliations:** Department of Respiratory Medicine, Huai'an Hospital of Traditional Chinese Medicine, Huai'an, 223001, China

## Abstract

**Objective:**

To explore the mechanism of action of Bu-Fei-Yi-Shen formula (BFYSF) in treating chronic obstructive pulmonary disease (COPD) based on network pharmacology analysis and molecular docking validation.

**Methods:**

First of all, the pharmacologically active ingredients and corresponding targets in BFYSF were mined by the Traditional Chinese Medicine Systems Pharmacology (TCMSP) database, the analysis platform, and literature review. Subsequently, the COPD-related targets (including the pathogenic targets and known therapeutic targets) were identified through the TTD, CTD, DisGeNet, and GeneCards databases. Thereafter, Cytoscape was employed to construct the candidate component-target network of BFYSF in the treatment of COPD. Moreover, the cytoHubba plug-in was utilized to calculate the topological parameters of nodes in the network; then, the core components and core targets of BFYSF in the treatment of COPD were extracted according to the degree value (greater than or equal to the median degree values for all nodes in the network) to construct the core network. Further, the Autodock vina software was adopted for molecular docking study on the core active ingredients and core targets, so as to verify the above-mentioned network pharmacology analysis results. Finally, the Omicshare database was applied in enrichment analysis of the biological functions of core targets and the involved signaling pathways.

**Results:**

In the core component-target network of BFYSF in treating COPD, there were 30 active ingredients and 37 core targets. Enrichment analysis suggested that these 37 core targets were mainly involved in the regulation of biological functions, such as response to biological and chemical stimuli, multiple cellular life processes, immunity, and metabolism. Besides, multiple pathways, including IL-17, Toll-like receptor (TLR), TNF, and HIF-1, played certain roles in the effect of BFYSF on treating COPD.

**Conclusion:**

BFYSF can treat COPD through the multicomponent, multitarget, and multipathway synergistic network, which provides basic data for intensively exploring the mechanism of action of BFYSF in treating COPD.

## 1. Introduction

Chronic obstructive pulmonary disease (COPD) is a common clinical disease characterized by respiratory system symptoms and flow limitation [1]. It is generally induced by airway and (or) alveolar abnormalities due to exposure to harmful particles and gases (like cigarette smoke), and its pathogenesis is mainly related to chronic airway and pulmonary inflammation, oxidative stress (OS), protease/antiprotease imbalance, and cell apoptosis [2–5]. COPD is associated with high morbidity and mortality rates and causes tremendous socioeconomic burdens, which has become an important public health issue [6]. In China, COPD is a major chronic respiratory system disease that severely impairs human health [7, 8].

Modern medicine can not attain satisfactory therapeutic effect or safety on stable COPD patients, and the limited efficacy must be maintained by long-term inhalation of bronchodilators and glucocorticoids [9–11]. By contrast, traditional Chinese medicine (TCM) exhibits obvious advantages in the treatment of COPD, which obviously improves the clinical symptoms of patients according to syndrome differentiation. In the TCM theory, COPD patients mostly suffer from syndrome of deficiency of both the lung and kidney. Therefore, tonifying the lung, strengthening the spleen, and boosting the kidney are the basic TCM therapeutic methods for COPD [12, 13]. Bu-Fei-Yi-Shen formula (BFYSF) is a cipher prescription for the treatment of COPD at Huai'an Hospital of Traditional Chinese Medicine, which is constituted by 6 Chinese herbal medicines, including Ginseng Radix et Rhizoma Rubra (Hongshen (HS)), Astragali Radix (Huangqi (HQ)), Epimedii Folium (Yinyanghuo (YYH)), Corni Fructus (Shanzhuyu (SZY)), Rehmanniae Radix (Dihuang (DH)), and Atractylodis Macrocephalae Rhizoma (Baizhu (BZ)). It has the effects of tonifying lung and spleen and warming and invigorating kidney yang. In clinic, BFYSF can effectively relieve the symptoms in COPD patients, improve the lung function, and enhance the patient exercise tolerance and quality of life. In addition, it can alleviate the cough severity, frequency, and expectoration amount and improve the wheezing symptom [14, 15], but its precise mechanism of action remains unclear so far.

Chinese medicine formula is the organic whole constituted by multiple traditional Chinese herbal medicines, which has a complex chemical composition [16]. Nonetheless, only partial chemical components possessing favorable pharmacokinetic properties can play therapeutic roles, and the therapeutic efficacy of a Chinese medicine formula may be derived from the joint action of multiple components [17]. Due to the complexity in chemical components of Chinese medicine and human body interaction, it is relatively difficult to illustrate the molecular mechanism of action of Chinese medicine formula in treating disease [18]. Network pharmacology is one of the key technical means to investigate the Chinese medicine formula in systems biology, which allows to reveal the pharmacological actions of herbal medicines and formulas, along with the molecular mechanisms, based on multidisciplinary integration, such as high-throughput omics, computer technology, pharmacology, and network database retrieval [19, 20]. This study applied the network pharmacology technology in predicting the pharmacodynamic material basis and molecular mechanism of BFYSF in treating COPD, and molecular docking technology was used for verification, so as to provide theoretical foundation for carrying out fundamental experiment study and reasonable clinical application of BFYSF.

## 2. Materials and Methods

### 2.1. Screening of Potential Pharmacodynamic Compounds and Related Targets in BFYSF

Using the Traditional Chinese Medicine Systems Pharmacology (TCMSP, Version: 2.3, https://tcmspw.com/index.php) database and the analysis platform [21], the names of six Chinese herbal medicines were input in succession to obtain the corresponding chemical compounds and related information. Then, the potential compounds in BFYSF were screened at the thresholds of bioavailability (OB) ≥ 30% and drug likeness (DL) ≥ 0.18 according to the literature reported method [22, 23]. Moreover, some compounds, such as astragaloside I, icariresinol, cornuside, rehmaglutin D, and atractylenolide I, which did not satisfy the criteria of bioavailability and DL values but were reported to possess extensive pharmacological activities or had high contents in single herbal medicine or were used as the identification compounds of single medicine in the Pharmacopeia [24–27], were also enrolled into the potential compounds of BFYSF. The potential targets of active ingredients were mined and integrated using the TCSMP and BATMAN-TCM databases (http://bionet.ncpsb.org/batman-tcm/) [28] according to the drug structural similarity evaluation and reverse molecular docking, for the construction of a potential target set of BFYSF.

### 2.2. Mining of COPD-Related Targets

The targets related to the COPD pathogenesis or therapeutics were obtained through retrieving the TTD database (http://db.idrblab.net/ttd/, Last update by June 1, 2020) [29], DisGeNet database (https://www.disgenet.org/, v7.0) [30], GeneCards database (https://www.genecards.org/, version: 5.0) [31], and CTD database (http://ctdbase.org/, Last update by June, 2020) [32] using the keyword “chronic obstructive pulmonary disease.” Specifically, drugs with abnormal status and the corresponding targets were eliminated in mining against the TTD database. In the DisGeNet database, the targets were ranked based on the disease specificity index (DSI) value from the highest to the lowest, and those greater than the median were selected. In the CTD database, the top 200 genes in terms of the Inference score were selected. In the GeneCards, targets with the score ≥ 10 were screened. Later, targets obtained from the above four databases were integrated to construct the COPD-related target set.

### 2.3. Construction of the Candidate and Core Component-Target Network of BFYSF in Treating COPD

First of all, the UniProt database (https://www.uniprot.org/) [33] was selected, the species was selected as “Homo sapiens,” and the names of targets obtained in the above two steps were standardized to gain the unique UniProt IDs and gene names. Subsequently, the two target sets were input, respectively, to the Venny tool (https://bioinfogp.cnb.csic.es/tools/venny/, version: 2.1) to acquire the common targets, which were the candidate targets of BFYSF in treating COPD. Thereafter, the Cytoscape software (version 3.7.2) [34] was employed to construct the candidate active component-target network of BFYSF in treating COPD, with “active component” being set as square, while “target” being set as circle. Then, the cytoHubba plug-in (version 0.1) [35] was employed to calculate and rank the degree of all nodes. Afterwards, nodes with degree greater than or equal to the median degree values for all nodes in the network (namely, the core active ingredients and core targets of BFYSF in treating COPD) were selected to establish the core component-target network. The node size in the core network was related to the degree value.

### 2.4. Verification of the Compound-Target Interactions

The interactions between compounds and targets were validated using the Autodock vina software (version: 1.1.2) [36]. The mol2 structure of compound was downloaded from the TCMSP database. In addition, the 3D structures of all target proteins were acquired based on the RCSB PDB database (http://www.rcsb.org/) [37]. The MOE approach and AutodockTools (version: 1.5.6) [38] were used to prepare ligands and proteins before molecular docking. For target protein, its crystal structure was subjected to pretreatments, which included water molecule removal, protonation and 3D hydrogenation, protein structural correction, energy optimization, and retention of target active region. Ligand structure should satisfy the low-energy conformation. The coordinates and box size for molecular docking were finalized according to the ligand location. To achieve a higher computational accuracy, we set the exhaustiveness parameter to 20. All other parameters were set as default values unless otherwise specified. Then, active ingredients were coupled into target protein in a semiflexible way, and 9 conformations were generated in total. The conformation with the highest affinity was selected as the final docking one.

### 2.5. GO-BP and KEGG Enrichment Analyses of Core Targets

To further explore the biological processes (BPs) and the regulatory signaling pathways involved in the core targets of BFYSF in treating COPD, the screened core target information was imported, and the Omicshare online software (https://www.omicshare.com/) was used for GO-BP and KEGG enrichment analyses of the targets at the threshold of *p* < 0.05.

## 3. Results

### 3.1. Screening of Potential Pharmacodynamic Components and Targets in BFYSF

Through TCMSP database retrieval, a total of 486 chemical components in the six herbal medicines constituting BFYSF were collected. Thereafter, the 486 components were screened at the active ingredient thresholds of OB ≥ 30% and DL ≥ 0.18, and 58 potential components were obtained. In addition, among those eliminated components, 13 compounds, which did not satisfy the inclusion criteria of bioavailability and DL values but were reported to possess extensive pharmacological activities or had high contents in single herbal medicine or were used as the identification compounds of single medicine in the pharmacopeia, were also enrolled as the potential components of BFYSF. Finally, HS, HQ, YYH, SZY, DH, and BZ had 10, 23, 2, 23, 4, and 13 active ingredients, respectively. Among them, beta-sitosterol and stigmasterol extensively existed in multiple herbal medicines. The basic information of the potential pharmacodynamic components of BFYSF is presented in Table S1.

Subsequently, the targets of these 71 potential components were retrieved against the TCMSP and BATMAN-TCM databases, and a total of 269 were identified (Table S2). As observed, HS, HQ, YYH, SZY, DH, and BZ had 77, 218, 21, 129, 42, and 34 potential targets, separately. There were obvious overlaps among those six herbal medicines, even though the target number of every herbal medicine was different. This suggested that the different components in BFYSF might show heterogeneous effects through regulating similar targets.

**Figure 1:**
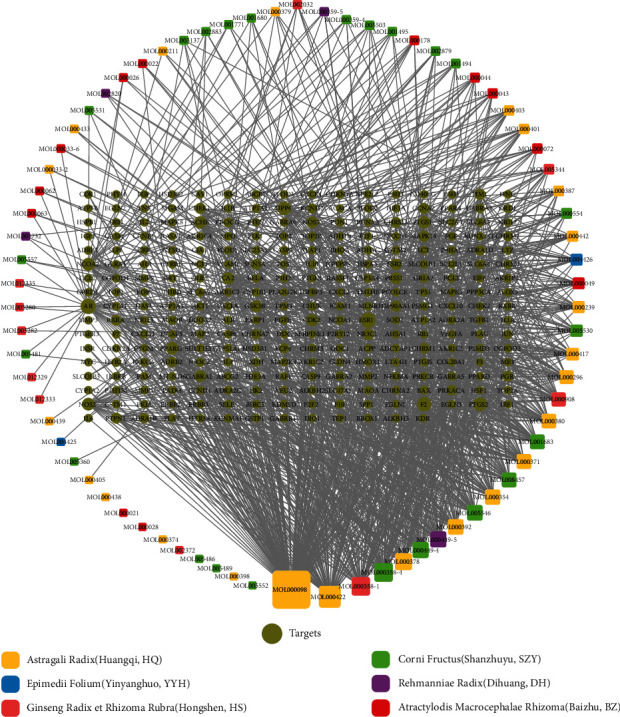
Construction of the BFYSF pharmacodynamic ingredient-target network. The active compounds (compounds ID) collected from different herbal medicines were linked with corresponding targets to construct the pharmacodynamic ingredient-target network, where a node indicates an active compound (the different colors of squares stand for different herbal medicines) and the target (green circles).

For the comprehensive understanding of the component-target network within BFYSF in a systemic and holistic manner, a network map was established using Cytoscape, which included 1147 edges along with 343 nodes (Figure 1). In the map, node degree was the number of targets or edges associated with the node according to topological analysis. Altogether 145 potential components were identified with the median ≥ 6 degrees from the constructed network. Among them, quercetin, kaempferol, beta-sitosterol, 7-O-methylisomucronulatol, stigmasterol, and formononetin exerted functions on 158, 72, 55, 47, 42, and 39 targets, respectively, which might be the significant active ingredients in BFYSF.

### 3.2. Mining of the Core Targets of BFYSF in Treating COPD

**Figure 2:**
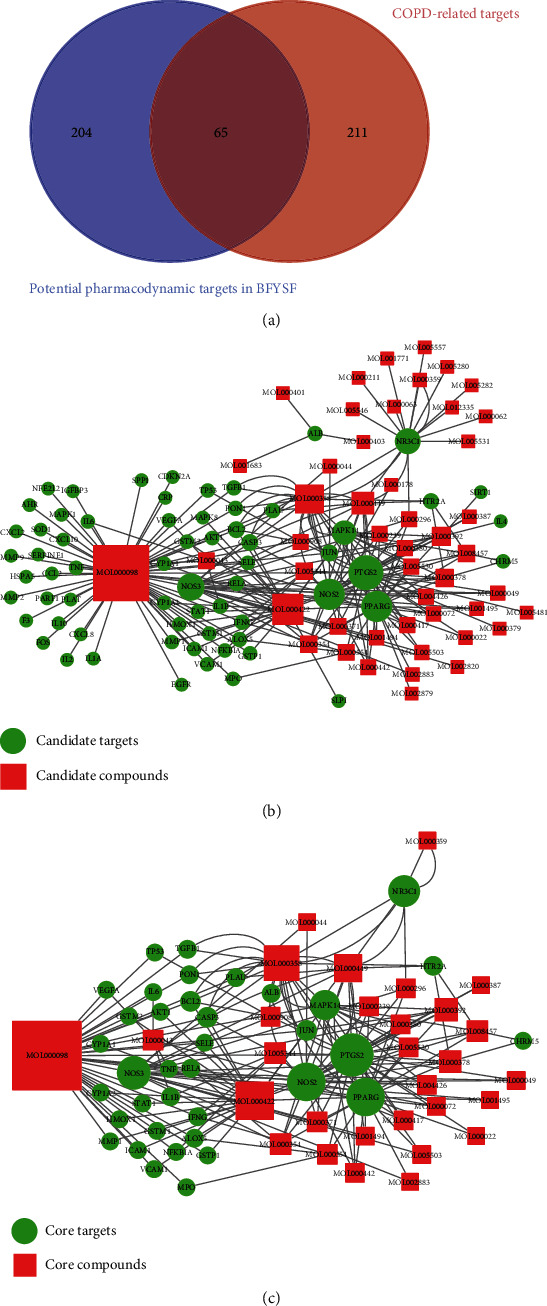
Excavation of core components and targets of BFYSF in treating COPD. (a) The Venn diagram showed that 65 potential targets in BFYSF were the same as the known pathological course-related targets of COPD. (b) The candidate component-target network of BFYSF in treating COPD. (c) The core component-target network of BFYSF in treating COPD.

COPD is a polygenic genetic disease, and its pathogenesis can be potentially illustrated by studying the gene-gene or gene-environment associations [39]. Through retrieving the TTD, DisGeNet, CTD, and GeneCards databases, altogether, 276 COPD-related targets (Table S3) were obtained. In addition, 65 out of the identified targets for the potential components of BFYSF were identified as the COPD- or therapeutic-related targets, suggesting that BFYSF showed certain therapeutic efficacy on COPD (Figure 2(a) and Table S4). These 65 targets were all candidate targets of BFYSF in treating COPD, while the corresponding active ingredients in BFYSF (*n* = 48) were the candidate active compounds of BFYSF.

Later, the Cytoscape software was employed to construct the candidate component-target network of BFYSF in treating COPD (Figure 2(b)). To further screen the core components and core targets of BFYSF in treating COPD, the cytoHubba plug-in was employed to calculate and sort the topological parameters (degree) of the nodes in the above-mentioned network (Table S5). Then, the median degree of all nodes was calculated, and nodes with values greater than or equal to the median (degree = 2) of all nodes were selected as the core components and core targets of BFYSF in treating COPD, respectively. Subsequently, the core component-target network (thirty-seven core targets and thirty core components) was constructed (Figure 2(c), Tables 1 and 2). Among all the core components, quercetin, kaempferol, beta-sitosterol, stigmasterol, and formononetin ranked the top five in terms of degree, which were derived from the HQ, HS, SZY, and DH in BFYSF, respectively. Some of them have been verified in previous studies to delay the course of COPD and improve symptoms [40, 41]. In addition, among all targets, PTGS2, PPARG, and NOS2 were the top three sorted by degree.

### 3.3. Verification of the Core Compound-Target Network

**Figure 3:**
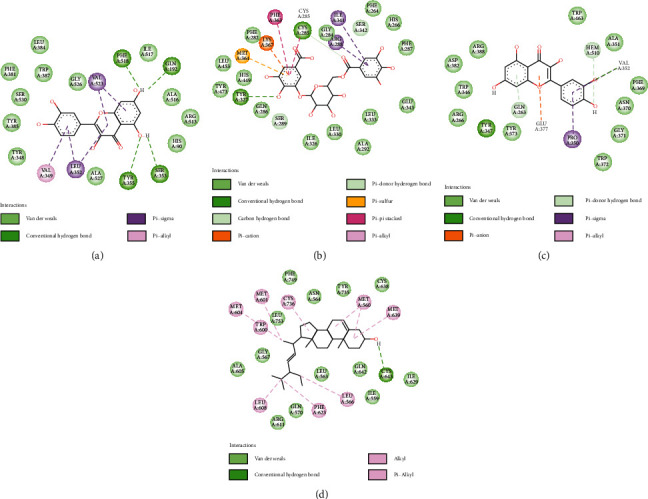
Virtual docking of core components and core targets of BFYSF in treating COPD. The virtual docking of quercetin with PTGS2 (a) and NOS2 (c), respectively. (b) The docking of PPARG with gallic acid-3-O-(6′-O-galloyl)-glucosid. (d) The virtual docking of stigmasterol with NRC31.

The associations of components with targets were assessed through molecular docking study, which reduced the network complexity and improved the accuracy. Virtual screening performed to determine the binding affinity between protein models and 9 core active compounds (the top three active ingredients in the order of degree value in the network and the outside core compounds with the top 1 degree value in each single Chinese herbal medicine constituting BFYSF), namely, atractylenolide I, quercetin, formononetin, kaempferol, beta-sitosterol, stigmasterol, gallic acid-3-O-(6′-O-galloyl)-glucoside, icariresinol, and ginsenoside rh2. Upon molecular docking, 4 targets (PTGS2, PPARG, NOS2, and NR3C1) together with 9 compounds were identified (Table 3 and Figure 3).

PTGS2, PPARG, NOS2, and NR3C1 were searched against the PDB protein database, separately, to obtain the 3D structures of 5kir, 3wmh, 4nos, and 4p6w proteins. As seen from the binding free energy results shown in Table 1, all the 9 core compounds had relatively tight bond to the 4 core targets. Among them, the compounds that showed most closely bound to PTGS2, PPARG, NOS2, and NRC31 were quercetin, gallic acid-3-O-(6′-O-galloyl)-glucoside, quercetin, and stigmasterol, respectively (Figure 3). Quercetin has been reported to decrease the expression and activity of COX-2 induced by ischemia reperfusion or arsenite [42, 43]. In addition, quercetin inhibited inflammatory response induced by LPS through iNOS/FAK/paxillin or p38/iNOS/NF-kappaB signaling pathway [44–46]. In the virtual docking (Figures 3(a) and 3(c)), quercetin was demonstrated to form hydrogen bonds with amino acid TYR^355^, PHE^518^, SER^353^, and GLN^192^ of COX-2 and VAL^352^, TYR^347^, and GLN^263^ of iNOS. At the same time, quercetin also formed a strong hydrophobic interaction with residues LEU^352^ and VAL^523^ of COX-2 and *π*-anion bonds with residues GLU^377^. The gallic acid-3-O-(6′-O-galloyl)-glucoside-PPAR*γ* complex might be stabilized through H-bond as well as *π*-cation bonds with CYS^285^, TYR^327^, SER^289^, and LYS^367^ (Figure 3(b)). Similarly, the stigmasterol-NR3C1 complex was stabilized by one H-bonds with residue CYS^643^ and hydrophobic interaction with residues MET^560^, CYS^736^, and LEU^566^ (Figure 3(d)).

### 3.4. Core Target Enrichment Analysis of BFYSF in Treating COPD

**Figure 4:**
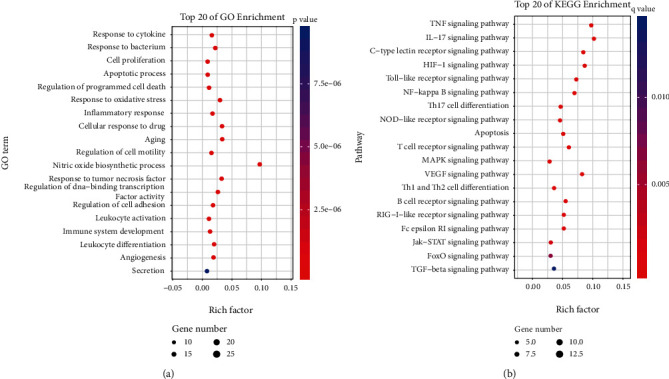
Omicshare-based enrichment analysis of core targets of BFYSF in treating COPD. The top twenty enriched GO-biological process (a) terms along with KEGG pathways (b) are shown. The abscissa corresponds to the GO terms or KEGG pathways, and the ordinate shows the enrichment factor. The color of the dot represents the adjusted *p* value/*q* value, and the size of the dot represents the number of core targets mapped to the reference GO terms or pathways.

To further understand the multitarget and multipathway mechanism of BFYSF in treating COPD, this study utilized the Omicshare online tool for the GO-PB and KEGG enrichment analyses of core targets, so as to mine the biological processes and signaling pathways involved in BFYSF in treating COPD (*p* < 0.05, FDR < 0.05). The most significantly enriched GO-BP items were response to biological and chemical stimuli, multiple cellular biological process, cell proliferation, immunity, metabolism, exercise, and biological regulation (Figure 4(a)). The above-mentioned core targets were mainly enriched into multiple KEGG pathways (Figure 4(b)), which revealed that these pathways exerted vital roles in BFYSF in treating COPD: (1) eleven immune-related pathways, including interleukin- (IL-) 17, FC*ζ*RI, RIG-I-like receptor, C-type lectin receptor, Th17 cell differentiation, NOD-like receptor, Toll-like receptor, T and B-cell receptor, Th1 and Th2 cell differentiation, and TNF; (2) four oxidative stress (OS) and inflammation related pathways, including the hypoxia-inducible factor 1 (HIF1), Fork head transcription factor O (FoxO), TNF, and JAK-STAT; (3) one protease/antiprotease imbalance related pathway, which was the transforming growth factor *β* (TGF-*β*) signaling pathway; (4) two pathways which were related to airway remodeling and airway mucus hypersecretion, including the MAPK and VEGF signaling pathway; and (5) one pathway which was related to cell growth and death, which was cell apoptosis.

## 4. Discussion

BFYSF is a cipher prescription used in the Department of Respiration at our hospital to treat COPD, which is most suitable for patients with lung and kidney deficiency. In the clinical treatment for COPD, BFYSF has significant therapeutic efficacy, but its active ingredients and mechanism remain unclear, which has hindered the further development and utilization of this prescription.

Network pharmacology is a new strategy of drug design and development proposed based on the rapid development of systems biology and polypharmacology. Hopkins first put forward this concept in 2007 [47]; in 2008, he proposed to transform the original new drug discovery pattern of “disease-single target-single drug” to the pattern of “disease-multiple targets-multiple drugs.” This thinking coincides with the “holistic view” in TCM [20, 48]. Therefore, applying the network pharmacology approach contributes to providing some research thinking for illustrating the BFYSF mechanism in the treatment of COPD.

It was discovered in this study that all the six single herbal medicines in this formula contained large amounts of components, and the components acted on multiple targets, or multiple components acted on the same target, which reflected the multicomponent and multitarget synergistic features of BFYSF. At the same time, the targets might be regulated by single herbal medicine or multiple herbal medicines of BFYSF, which showed that in a formula, both the unique contribution of single herbal medicine and the synergy of multiple herbal medicines existed. As discovered from the component-target network of BFYSF in treating COPD, 48 components in this prescription might act on 65 targets to exert the therapeutic effect. Furthermore, based on the topological parameters of nodes in the component-target network, it was speculated that PTGS2, PPARG, NOS2, and NR3C1 were the core targets for BFYSF in treating COPD. Besides, the interactions between core components and core targets were verified through molecular docking study. The subsequent GO-BP and KEGG enrichment analyses revealed that these targets mainly participated in regulating inflammatory immune response, OS, suppressing protease/antiprotease imbalance, improving airway remodeling and airway mucus hypersecretion, energy metabolism, and modulating the related signaling pathways.

Airway and pulmonary inflammation, protease/antiprotease imbalance, and oxidation/antioxidation imbalance are the major pathogenic mechanisms of COPD. As suggested in research, the Th17 cell proportion and COX-2 expression in the lung tissue and peripheral blood of COPD patients significantly increase, the inflammatory cytokine TNF-*α* levels in blood and airway elevate, the neutrophil elastase level in serum elevates, *α*1-AT level decreases, and the protease/antiprotease balance is destroyed [49–53]. In the COPD model mice induced by cigarette smoke stimulation, the body produces OS response, which then activates the Nrf2 nuclear transcription and suppresses its target gene HOMX1 expression [54]. Regulating the SIRT3/FoxO pathway and suppressing HIF-1 expression and release can improve the inflammatory and OS statuses in COPD [55, 56]. It is reported in plenty of articles that multiple core active ingredients of BFYSF in treating COPD can treat COPD through improving airway and pulmonary inflammation or the antioxidant activity. For instance, isorhamnetin can suppress the TNF-induced airway inflammatory response; astragaloside and gallic acid can alleviate inflammation to suppress the cigarette-induced COPD; and quercetin and kaempferol can treat COPD through its antioxidation and anti-inflammatory activities [41, 57].

Airway remodeling and airway mucus hypersecretion are the major pathological features of COPD, which are mainly manifested as airway wall structural changes, luminal stenosis, and flow limitation [58, 59]. Research suggests that MAPK activation is related to the airway remodeling in COPD [60]. Reducing the VEGF and COX-2 expression levels in COPD rats can suppress airway remodeling, thus improving COPD [61, 62]. PPAR activation can inhibit the inflammatory and OS reactions and participate in regulating the mucus hypersecretion in COPD patients [63]. Quercetin, the core ingredient in BFYSF, has been verified to improve pulmonary function and treat COPD through protecting from airway remodeling.

## 5. Conclusion

To sum up, the mechanism of action by which BFYSF treats COPD may be related to the regulation of immune inflammatory response, antioxidation, suppression of protease/antiprotease imbalance, and improvement of airway remodeling and airway mucus hypersecretion.

## Figures and Tables

**Table 1:** Degree values of core targets for BFYSF against COPD.

Targets name	Degree
PTGS2	29
PPARG	24
NOS2	24
NOS3	19
NR3C1	17
MAPK14	15
JUN	5
CASP3	5
BCL2	5
HTR2A	4
TGFB1	3
RELA	3
PON1	3
PLAU	3
IL1B	3
IFNG	3
ALOX5	3
ALB	3
VEGFA	2
VCAM1	2
TP53	2
TNF	2
STAT1	2
SELE	2
NFKBIA	2
MPO	2
MMP1	2
IL6	2
ICAM1	2
HMOX1	2
GSTP1	2
GSTM2	2
GSTM1	2
CYP1A2	2
CYP1A1	2
CHRM5	2
AKT1	2

**Table 2:** 30 core pharmacologically active ingredients of BFYSF in the treatment of COPD.

Compound no.	Degree in core network	Compound name
MOL000098	57	Quercetin
MOL000422	24	Kaempferol
MOL000358	21	Beta-sitosterol
MOL000449	13	Stigmasterol
MOL000392	8	Formononetin
MOL000378	7	7-O-Methylisomucronulatol
MOL000354	6	Isorhamnetin
MOL005344	6	Ginsenoside rh2
MOL008457	6	Tetrahydroalstonine
MOL000043	5	Atractylenolide I
MOL000371	5	3,9-Di-O-methylnissolin
MOL000554	5	Gallic acid-3-O-(6′-O-galloyl)-glucoside
MOL000239	4	Jaranol
MOL000296	4	Hederagenin
MOL000380	4	(6aR,11aR)-9,10-Dimethoxy-6a,11a-dihydro-6H-benzofurano[3,2-c]chromen-3-ol
MOL000417	4	Calycosin
MOL000442	4	1,7-Dihydroxy-3,9-dimethoxy pterocarpene
MOL000908	4	Beta-elemene
MOL004426	4	Icariresinol
MOL005530	4	Hydroxygenkwanin
MOL000049	3	3*β*-Acetoxyatractylone
MOL000022	3	14-Acetyl-12-senecioyl-2E,8Z,10E-atractylentriol
MOL000044	3	Atractylenolide II
MOL000072	3	8*β*-Ethoxy atractylenolide III
MOL000359	2	Sitosterol
MOL000387	2	Bifendate
MOL001494	2	Mandenol
MOL001495	2	Ethyl linolenate
MOL002883	2	Ethyl oleate (NF)
MOL005503	2	Cornudentanone

**Table 3:** Virtual docking of core bioactive ingredients and core targets for BFYSF in treating COPD.

Core ingredients	Binding energy/(kcal mol^−1^)			
	PTGS2	PPARG	NOS2	NR3C1
MOL000043	-7.2	-6.9	-7.8	-7.8
MOL000098	-9.6	-8.4	-9.1	-8.4
MOL000358	-4.8	-5.4	-6.9	-9.3
MOL000392	-7.3	-8.3	-8.3	-7.2
MOL000422	-9.5	-8.3	-8.6	-8.2
MOL000449	-5.2	-6.2	-7.4	-9.5
MOL000554	-6	-8.7	-9	-7.8
MOL004426	-6	-6.8	-7.5	-7.6
MOL005344	-8.9	-6.2	-7.5	-4.3

## Data Availability

The datasets used and/or analyzed during the current study are available from the corresponding author on reasonable request.

## Supplementary Materials

Supplementary 1 Table S1: all the pharmacodynamic ingredients of BFYSF.

Supplementary 2 Table S2: all the potential pharmacodynamic targets of BFYSF.

Supplementary 3 Table S3: known COPD-related targets.

Supplementary 4 Table S4: BFYSF shared 65 potential pharmacodynamic targets with known COPD-related targets.

Supplementary 5 Table S5: degree values of nodes in the candidate active ingredient-target network of BFYSF in treating COPD.
